# Physical behaviour profiles and their associations with fitness and function in older adults: a cross-sectional latent profile analysis

**DOI:** 10.1186/s11556-025-00397-4

**Published:** 2026-01-12

**Authors:** Vera Zymbal, João P. Magalhães, Fátima Baptista, Gil B. Rosa, Eduardo B. Cruz, Luís B. Sardinha

**Affiliations:** 1 Instituto Politécnico de Setúbal, Escola Superior de Educação, Centro de Investigação em Qualidade de Vida (CIEQV), Setúbal, Portugal; 2 CIPER, Faculdade de Motricidade Humana, Universidade de Lisboa, Lisbon, Portugal; 3 Instituto Politécnico de Setúbal, Escola Superior de Saúde, Setúbal, Portugal

**Keywords:** Ageing, Physical activity, Sedentary behaviour, Latent profile analysis, Strength, Endurance, Agility, Flexibility

## Abstract

**Background:**

Traditional variable-centred approaches often analyse physical behaviours (sedentary behaviour [SB], light physical activity [LPA], and moderate-to-vigorous physical activity [MVPA]) in isolation, potentially masking their combined effects on outcomes. This study applied latent profile analysis, a person-centred approach, to identify naturally occurring physical behaviour profiles in older adults and examined their associations with physical fitness and physical function.

**Methods:**

This cross-sectional study included 1,095 older Portuguese adults (≥ 65 years; 765 females). SB, LPA, and MVPA were assessed using accelerometry (Actigraph; Pensacola, Florida) on the right hip and expressed as percentages of waking time. Latent profile analysis was used to identify distinct profiles based on these percentages. Physical fitness was evaluated by Senior Fitness Test battery and handgrip strength. Physical function was assessed using the 12-item Composite Physical Function questionnaire. Generalised linear models, adjusted for age, were used to examine associations between profiles and outcomes.

**Results:**

Three distinct profiles emerged for both sexes: “balanced movers” (~ 50% SB, ~ 46% LPA, ~ 4% MVPA), “intermediate movers” (~ 66% SB, ~ 32% LPA, ~ 2% MVPA), and “highly sedentary” (~ 80% SB, ~ 20% LPA, < 1% MVPA). Compared to the “highly sedentary” groups, both “balanced movers” and “intermediate movers” demonstrated better performance on most physical fitness tests and reported higher physical function. Notably, “intermediate movers”, performed similarly to “balanced movers” in most measures.

**Conclusions:**

Distinct physical behaviour profiles exist among older Portuguese adults. Profiles characterised by lower SB and higher LPA, even when not fully meeting MVPA recommendations (“intermediate movers”), were associated with better physical fitness and physical function compared to the “highly sedentary” profile. This underscores the importance of reducing SB and promoting LPA along with MVPA. By uncovering these behavioural profiles among older adults, latent profile analysis provides valuable insights to guide the development of more personalized interventions for healthy ageing.

**Supplementary Information:**

The online version contains supplementary material available at 10.1186/s11556-025-00397-4.

## Background

 Maintaining physical fitness and physical function is essential for older adults, as it plays a key role in preserving their physical independence [1]. However, as individuals age, most experience a decline in physical fitness components (e.g., cardiorespiratory endurance, muscular strength, flexibility, agility), impacting physical function – the ability to perform activities of daily living (ADLs) [2]. This decline varies among individuals and is influenced by lifestyle factors, particularly physical activity and sedentary behaviour (SB) [3–6]. Despite the established benefits of physical activity [7–9], particularly moderate-to-vigorous physical activity (MVPA), many older adults do not meet the recommended guidelines and spend substantial time in SB [10], potentially accelerating functional decline [11].

The growing body of evidence demonstrating the detrimental effects of SB on health outcomes across the lifespan has prompted updates to the World Health Organisation physical activity guidelines. The most recent revision not only reaffirms the importance of MVPA for older adults, but also emphasizes the need to reduce SB and incorporate physical activity of all intensities, including light-intensity physical activity (LPA), into daily routines [12]. This reflects a growing understanding of the 24-hour activity spectrum and the interdependence of these behaviours; time spent in one behaviour directly impacts time available for others [13]. Furthermore, emerging evidence suggests that different combinations or patterns of these behaviours, rather than single behaviours in isolation, may differentially influence health outcomes [14, 15].

Traditionally, research has employed variable-centred approaches that primarily focus on associations between single variables (e.g., MVPA or SB) and health outcomes [13]. While valuable, these methods may not fully capture the synergistic, additive, or counteracting effects of co-occurring behaviours, or identify distinct subgroups within the population characterised by unique behavioural patterns. Such limitation hinders a comprehensive understanding of how the interplay between SB, LPA, and MVPA collectively impacts health. In response to these limitations, person-centred approaches, such as latent profile analysis, have gained traction [13, 16]. Latent profile analysis allows researchers to identify unobserved subgroups (latent profiles) of individuals based on shared patterns across multiple behavioural variables. This approach offers a more holistic view by considering how SB, LPA, and MVPA combine within individuals [16]. Several studies have successfully applied latent profile analysis to understand a wide range of physical behaviour patterns in adult populations, linking distinct profiles to various health outcomes including cognitive function [17], metabolic risk [18], quality of life [19], and intrinsic capacity [20]. These studies highlight the utility of latent profile analysis in revealing nuanced relationships often overlooked by variable-centred methods and its potential for informing tailored interventions. However, to the best of our knowledge there are no studies that examining accelerometry measured physical behaviour profiles (SB, LPA, MVPA) and their specific associations with physical fitness and physical function in older adults. By adopting a latent profile analysis approach, this study addresses methodological gaps highlighted in recent consensus statements [13] and contributes to a more nuanced understanding of how distinct physical behaviour patterns are associated with age-related physical health indicators in older adults. Therefore, this study aimed to: (1) identify distinct profiles of physical behaviour (based on accelerometer-measured SB, LPA, and MVPA) among Portuguese older adults using latent profile analysis and (2) investigate how these profiles are associated with comprehensive measures of physical fitness and physical function.

## Materials and methods

### Participants

This study employed a cross-sectional design using data from a national representative sample of the Portuguese physical activity and sports monitoring system collected between 2017 and 2018. A detailed description of the data collection is available in a prior publication [10]. In brief, the sample was selected by proportionate stratified random sampling considering the number of people by age and gender in each region of mainland Portugal (Alentejo, Algarve, Center, Lisbon Metropolitan Area, and North). The participants were recruited from community senior (social) centres, public and private institutions, sports clubs, and social/sports events across the country. This report includes only older adults (> = 65 years; age range: 65–98 years) with complete and valid accelerometry data, (*n* = 1095, 765 females). The study was conducted in accordance with the Declaration of Helsinki, and approved by the Ethics Committee of the Faculty of Human Kinetics, University of Lisbon (number: 25/2020). Informed consent was obtained from all subjects involved in the study.

### Anthropometry

Body weight was assessed with the participants barefoot and with light clothes on an electronic scale to the nearest 0.1 kg, while height was assessed with a standard stadiometer in centimetres (Seca, Hamburg, Germany). Body mass index (BMI) was determined by taking the ratio between body weight (kg) and the squared height (m^2^).

### Physical fitness and physical function

Physical fitness was assessed through the Senior Fitness Test Battery [21] which includes six validated tests for evaluating physiological parameters supporting mobility and independence in older adults. The 30-s chair-stand, arm-curl, chair sit-and-reach, back-scratch, 8-ft up-and-go, and 6-min walk tests measured lower and upper body strength, lower and upper body flexibility, agility, and functional endurance, respectively. A detailed description of each test protocol is available elsewhere [21]. Muscle strength was also evaluated through handgrip (Hydraulic Hand Dynamometer, Jamar). Participants performed the test in a standing position with their arms alongside the body, holding the dynamometer. The assessment was first carried out with the dominant hand and subsequently with the non-dominant hand. Each hand was tested three times in an alternating order, and the mean value for each hand was computed. The highest mean value was retained for the analysis. To account for body size differences, handgrip strength was adjusted by dividing the values by the square of height (kg/m²) [22]. Physical function, was assessed subjectively by the 12-item Composite Physical Function (CPF) scale [23, 24]. This self-report questionnaire was designed to assess an individual’s physical function across a range of abilities, including basic ADLs (e.g., dressing oneself), instrumental ADLs (e.g., housework), and advanced activities (e.g., more vigorous exercise activities). Each item is rated on a 3-category scale (0 = cannot do, 1 = can do with help, 2 = can do independently) with the total providing an overall measure of physical function.

### Sedentary behaviour and physical activity

SB, LPA and MVPA were evaluated by accelerometry (min·day^− 1^). Participants wore an accelerometer (Actigraph GT3X or GT1M; Pensacola, Florida) on the right hip, near the iliac crest for 7 consecutive days, removing it only for sleeping or water-based activities. Movement data from both accelerometers were collected in 15-s epochs (Actilife v.6.9.1). Days with wear time greater than 10 h were classified as valid. Participants were included in the analysis only if they had a minimum of three valid days, with at least one occurring on a weekend [25]. Physical activity intensities and SB cut points were defined according to Troiano et al. [25]. To account for variability in wear time, physical activity and SB were expressed as percentage of wear time, calculated as the average minutes spent in each intensity (min·day⁻¹) divided by the average wear time (min·day⁻¹), multiplied by 100.

### Statistical analysis

Latent profile analysis was conducted using the snowRMM module (v.5.5.7) [26], of Jamovi software (version 2.3.28) to identify distinct subgroups (profiles) of participants based on their average percentage of waking time spent in SB, LPA, and MVPA. Latent profiles of physical behaviour were computed separately for females and males due to the observed sex differences in physical activity variables, as well as differences in physical fitness and physical function - females engaged more in LPA while males spent more time in MVPA (with no differences in SB), and males outperformed females in most physical fitness tests and reported better physical function while females showed superior flexibility (data not shown). Models specifying 2 to 6 potential profiles were estimated with varying variances and covariances fixed to 0. The optimal number of profiles was determined based on a combination of model fit indices, interpretability, and theoretical relevance. Since the fit indices may continue to improve as more profiles are extracted, especially in larger samples, we created elbow plots that represented the gains associated with additional profiles and identified the point at which the improvement in model fit plateaued.

To analyse differences in participant characteristics across the identified physical behaviour profiles for females and males, generalised linear models were applied according to the distributional properties of each variable. Model fit and assumptions were assessed using appropriate goodness-of-fit indices and diagnostic evaluation of residuals. All variables were presented as estimated marginal means (EMMs) with standard errors (SE) and 95% confidence intervals (CI). Variables of physical behaviour—specifically SB, LPA, and MVPA, in minutes per day—were adjusted for accelerometry wear time. Post-hoc analyses were conducted using Holm-Bonferroni correction to control for multiple comparisons and reduce the risk of type I error.

To assess the associations between physical behaviour profiles and outcomes (physical fitness and physical function), generalised linear models were employed, adjusting for age. The profile was treated as an independent variable and physical fitness and physical function measures were treated as dependent variables. For continuous dependent variables, linear models with an identity link function were used. Completion time variables were analysed using inverse gaussian models with a log link function. For dependent count variables, Poisson models with a log link function were applied. These models were chosen based on the nature and distribution of the dependent variables and the model’s fit indices (i.e., Akaike Information Criterion (AIC) and Bayesian Information Criterion (BIC)) as well as on the model’s residual distribution. Post-hoc analyses were conducted using Holm-Bonferroni correction to control for multiple comparisons and reduce the risk of type I error. Marginal means were estimated to examine the expected values of the dependent variables for each profile of physical behaviour adjusted for age. For all analysis, the significance level was set at *p* < 0.05.

## Results

### Identified physical behaviour profiles and their characteristics

**Fig. 1:**
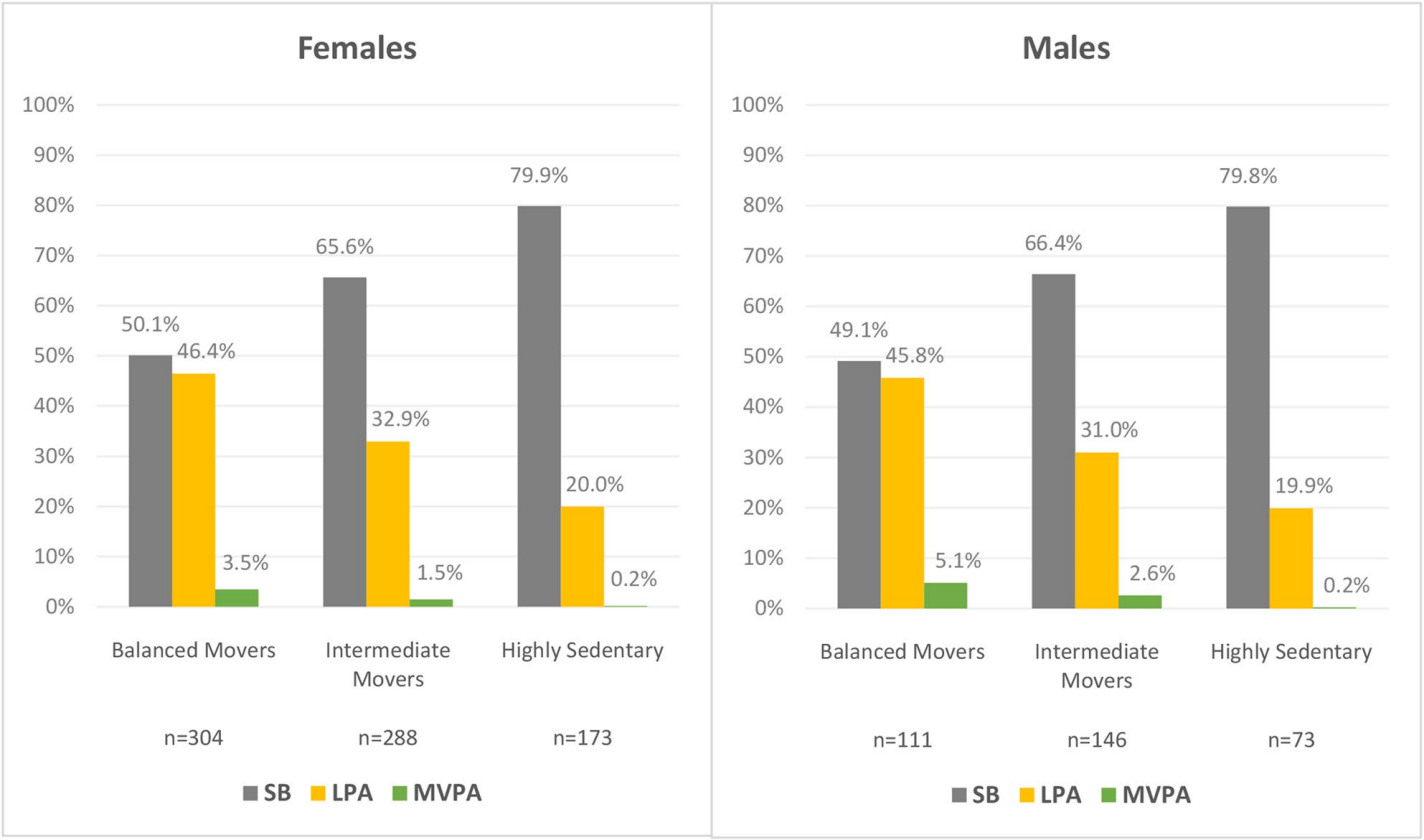
Profiles of physical behaviour for females and males, showing the proportions of *SB* Sedentary behaviour, *LPA* Light physical activity, and MVPA Moderate-to-vigorous physical activity

Latent profile analysis, performed separately within females and males, identified three distinct profiles of physical behaviour in each sex, which were designated as “balanced movers,” “intermediate movers,” and “highly sedentary.” These profiles were characterized by significant differences in the proportions of SB, LPA, and MVPA. Fit indices supporting the 3-profiles (ranging from two to six profiles) and the elbow plot illustrating the improvements across models are provided in the supplementary material for both females (Table S1 and Figure S1) and males (Table S2 and Figure S2). These plots indicate that the improvement in model fit plateaus at three profiles of physical behaviour for both sexes. Figure 1 presents the profiles identified for females and males, illustrating the average proportions of SB, LPA, and MVPA spent throughout the day. The corresponding daily values of physical activity and SB, expressed in minutes per day, are summarized in Table 1.

#### Balanced movers

This profile included 304 females and 111 males, who exhibited the most balanced distribution of physical behaviours, spending approximately half of waking time in SB (females: 50.1%, ~ 06h34 min/day; males: 49.1%, ~6h26 min/day) and half in physical activity (females: 49.9% [46.4% LPA, ~6h07min/day and 3.5% MVPA, ~ 27 min/day]; males: 50.9% [45.8% LPA, ~6h05min/day and 5.1% MVPA, ~ 40 min/day]. Notably, this group had the highest proportion engaging in MVPA and was, on average, the only profile meeting the World Health Organization MVPA recommendation (≥ 21.4 min per day), with 52.3% of females and 67.6% of males meeting this benchmark.

#### Intermediate movers

This profile included 288 females and 146 males, who exhibited higher SB (females: 65.6%, ~8h38min/day; males: 66.4%, ~8h45min/day) and lower total physical activity compared to “balanced movers” (females: 34.4% [32.9% LPA, ~4h19min/day and 1.5% MVPA, ~ 11 min/day]; males: 33.6% [31.0% LPA, ~4h06min/day, and 2.6% MVPA, ~ 21 min/day]). Although for males in this group, MVPA averaged 21 min per day, just shy of the recommended 21.4 min per day, only a minority (39.73% of males) achieved this target. For females, MVPA averaged ~ 11 min/day with only 17.36% of females in this group achieving the MVPA recommendation.

#### Highly sedentary

This profile, with 173 females and 73 males, was marked by overwhelmingly sedentary pattern. SB dominated waking hours (females: 79.9%, ~ 10h21min/day; males: 79.8%, ~ 10h25min/day). Total physical activity was the lowest of all profiles (females: 20.2%, ~2h48min/day; males: 20.2%, ~2h47min/day) with minimal time in LPA and negligible MVPA (females: 20.0% LPA, ~2h46min/day, 0.2% MVPA, ~2 min/day; males 19.9% LPA, ~2h43min/day, 0.2% MVPA ~ 3 min/day). None of the participants in this group met the recommended MVPA levels.

The characteristics of the participants categorized by the sex-specific physical behaviour profiles are presented in Table 1 as estimated marginal means (EMMs) with standard errors (SE) and 95% confidence intervals (95% CI).

Among females, those classified as “Balanced Movers” were the youngest (71.55 ± 0.33 yrs), followed by the “Intermediate Movers” (75.17 ± 0.36 yrs), who were older than the “Balanced Movers” but younger than the “Highly Sedentary” group (81.76 ± 0.50 yrs). Regarding body mass, no significant differences were observed among the female behavioural profiles (65.95 ± 0.64 kg, 67.35 ± 0.66 kg, and 66.80 ± 0.85 kg for “Balanced Movers”, “Intermediate Movers”, and “Highly Sedentary”, respectively). Nevertheless, “Highly Sedentary” females were shorter (151.08 ± 0.49 cm) than both “Balanced Movers” (153.40 ± 0.37 cm) and “Intermediate Movers” (152.99 ± 0.38 cm). They also showed a higher BMI (29.23 ± 0.34 kg/m2) than “Balanced Movers” (28.02 ± 0.25 kg/m2).

In males, age did not differ between the “Balanced Movers” (73.26 ± 0.61 yrs) and “Intermediate Movers” (74.90 ± 0.54 yrs); however, the “Highly Sedentary” group included the oldest individuals (80.77 ± 0.82 yrs) compared with both the “Balanced Movers” and “Intermediate Movers” profiles. No significant differences were found across male profiles for any of the anthropometric measures (body mass, height, or BMI).

The physical activity and SB variables, expressed in minutes per day, were described within the physical behaviour profiles as complementary information to Figure 1 (Table 1).

**Table 1:** Participant characteristics and daily physical activity and sedentary behaviour by sex-specific physical behaviour profiles

*Females *	**"Balanced Movers" n=304 (39.7%)**			**“Intermediate Movers" n=288 (37.6%)**			**"Highly Sedentary" n=173 (22.6%)**		
	**EMM**	**SE**	**95% CI**	**EMM**	**SE**	**95% CI**	**EMM**	**SE**	**95% CI**
Age (yrs)	71.55 ^b,c^	± 0.33	70.89; 72.20	75.17 ^a,c^	± 0.36	74.47; 75.87	81.76 ^a,b^	± 0.50	80.77; 82.74
BMI (Kg/m^2^)	28.02 ^c^	± 0.25	27.54; 28.51	28.75	± 0.26	28.24; 29.26	29.23^a^	± 0.34	28.56; 29.90
Body mass (Kg)	65.95	± 0.64	64.69; 67.22	67.35	± 0.66	66.05; 68.65	66.80	± 0.85	65.13; 68.48
Body height (cm)	153.40 ^c^	± 0.37	152.68; 154.12	152.99 ^c^	± 0.38	152.25; 153.73	151.08 ^a,b^	± 0.49	150.12; 152.03
SB (min·day^−1^)	393.84 ^b,c^	± 2.97	388.01; 399.67	518.02 ^a,c^	± 3.04	512.04; 523.99	620.78 ^a,b^	± 4.01	612.91; 628.65
LPA (min·day^−1^)	367.47 ^b,c^	± 3.14	361.29; 373.64	259.35 ^a,c^	± 3.22	253.03; 265.67	165.55 ^a,b^	± 4.24	157.22; 173.88
MVPA (min·day^−1^)	27.48 ^b,c^	± 0.94	25.64; 29.32	11.42 ^a,c^	± 0.96	9.54; 13.31	2.46 ^a,b^	± 1.27	−0.03; 4.94

	**"Balanced Movers" n=111 (33.6%)**			**“Intermediate Movers" n=146 (44.2%)**			**"Highly Sedentary"n=73 (22.1%)**		
*Males *	**EMM**	**SE**	**95% CI**	**EMM**	**SE**	**95% CI**	**EMM**	**SE**	**95% CI**
Age (yrs)	73.26^c^	± 0.61	72.07; 74.45	74.90^c^	± 0.54	73.84; 75.97	80.77 ^a,b^	± 0.82	79.15; 82.39
BMI (Kg/m^2^)	27.58	± 0.39	26.81; 28.35	27.40	± 0.34	26.73; 28.07	28.07	± 0.49	27.10; 29.04
Body mass (Kg)	76.33	± 1.25	73.86; 78.79	75.91	± 1.09	73.77; 78.05	75.53	± 1.53	72.52; 78.54
Body height (cm)	166.21	± 0.63	164.96; 167.46	166.20	± 0.55	165.11; 167.28	163.95	± 0.77	162.43; 165.47
SB (min·day^−1^)	385.69 ^b,c^	± 4.95	375.94; 395.43	524.58 ^a,c^	± 4.24	516.23; 532.93	624.62 ^a,b^	± 6.08	612.67; 636.58
LPA (min·day^−1^)	365.31 ^b,c^	± 5.13	355.22; 375.39	245.73 ^a,c^	± 4.39	237.09; 254.37	163.58 ^a,b^	± 6.29	151.20; 175.95
MVPA (min·day^−1^)	40.54 ^b,c^	± 2.23	36.16; 44.92	21.22 ^a,c^	± 1.91	17.47; 24.98	3.33 ^a,b^	± 2.73	−2.04; 8.70

*Abbreviations*: *EMM* Estimated marginal mean, *SE* Standard error, *CI* Confidence intervals, *BMI* Body mass index, *SB* Sedentary behaviour, *LPA* Light physical activity, *MVPA* Moderate-to-vigorous physical activity

Within each sex, differences between the physical behaviour profiles were tested using Generalised Linear Models with Holm-Bonferroni correction for multiple post-hoc comparisons

SB, LPA, and MVPA were adjusted by wear time, Superscripts a, b, c denote significant pairwise differences

The significance level was set at p < 0.05

ᵃ difference from "Balanced Movers" profile

^b^ difference from "Intermediate Movers" profile

^c^ difference from "Highly Sedentary" profile

### Physical fitness and physical function outcomes

Tables 2 and 3 present the estimated parameters and estimated marginal means (EMMs), respectively, derived from the generalised linear models, adjusted for age.

#### Females

For females, the analysis revealed that physical behaviour profiles were significantly associated with most physical fitness and physical function outcomes, with the exception of handgrip strength (Table 2). In general, females in the “Balanced Movers” and “Intermediate Movers” profiles had superior physical fitness and physical function compared to their “Highly Sedentary” counterparts. Specifically, lower body strength was significantly greater, with “Balanced Movers” performing 1.38 times, or 38% (RR = 1.38, *p* < 0.001) and “Intermediate Movers” performing 1.36 times, or 36% (RR = 1.36, *p* < 0.001) more chair stand repetitions than the “Highly Sedentary” group (Table 2). These differences correspond to EMMs of 14.18 and 13.94 repetitions for the “Balanced Movers” and “Intermediate Movers”, respectively, compared to 10.28 repetitions for the sedentary group (Table 3). Similarly, upper body strength was greater, as “Balanced Movers” completed 1.32 times (RR = 1.32, *p* < 0.001) and “Intermediate Movers” 1.28 times (RR = 1.28, *p* < 0.001) more arm curl repetitions (Table 2). This translated into mean values of 17.07 and 16.55 repetitions, respectively, versus 12.92 for “Highly Sedentary” females (Table 3). Flexibility was also markedly higher; “Balanced Movers” showed an advantage of 10.11 cm (β = 10.11, *p* < 0.001) in upper body and 7.56 cm (β = 7.56, *p* < 0.001) in lower body flexibility, while “Intermediate Movers” showed an advantage of 9.48 cm (β = 9.48, *p* < 0.001) and 6.68 cm (β = 6.68, *p* < 0.001), respectively, compared to the “Highly Sedentary” group (Table 2). Furthermore, agility was substantially better, as “Balanced Movers” and “Intermediate Movers” completed the 8-Foot Up and Go test 43% (TR = 0.57, *p* < 0.001) and 41% (TR = 0.59, *p* < 0.001) faster, respectively (Table 2). Their mean completion times were 7.14 s and 7.46 s, in contrast to 12.55 s for the highly sedentary group (Table 3). Functional endurance was also higher, with “Balanced Movers” walking 179.52 m farther (β = 179.52, *p* < 0.001) and “Intermediate Movers” walking 122.45 m farther (β = 122.45, *p* < 0.001) than “Highly Sedentary” females (Table 2). Finally, their composite physical function (CPF) scores were 5.72 points (β = 5.72, *p* < 0.001) and 3.89 points (β = 3.89, *p* < 0.001) higher, respectively (Table 2), corresponding to EMMs of 19.38 and 17.55 versus 13.66 for the “Highly Sedentary” females (Table 3). Notably, significant differences between the “Balanced Movers” and “Intermediate Movers” were only observed for functional endurance (β = 57.07, *p* < 0.001) and the CPF score (β = 1.83, *p* < 0.001), where “Balanced Movers” showed better performance (Table 2).

**Table 2:** Associations of physical behaviour profiles with physical fitness and physical function outcomes in females and males

		Females			Males		
		β	SE	*p*	β	SE	*p*
Handgrip (Kg/m^2^)	Balanced movers - Intermediate movers	0.06	0.24	1.000	−0.29	0.46	0.532
	Balanced movers - Highly sedentary	0.30	0.32	1.000	1.15	0.59	0.103
	Intermediate movers - Highly sedentary	0.24	0.30	1.000	1.43	0.54	0.026
		RR			RR		
Chair Stand (rep/30 s)	Balanced movers - Intermediate movers	1.02	0.02	0.472	1.00	0.03	0.922
	Balanced movers - Highly sedentary	1.38	0.05	< 0.001	1.42	0.07	< 0.001
	Intermediate movers - Highly sedentary	1.36	0.05	< 0.001	1.42	0.07	< 0.001
		RR			RR		
Arm Curl (rep/30 s)	Balanced movers - Intermediate movers	1.03	0.02	0.154	1.05	0.03	0.102
	Balanced movers - Highly sedentary	1.32	0.04	< 0.001	1.37	0.06	< 0.001
	Intermediate movers - Highly sedentary	1.28	0.04	< 0.001	1.31	0.06	< 0.001
		β			β		
Back Scratch (cm)	Balanced movers - Intermediate movers	0.63	1.09	0.562	−2.61	1.79	0.146
	Balanced movers - Highly sedentary	10.11	1.47	< 0.001	5.37	2.35	0.046
	Intermediate movers - Highly sedentary	9.48	1.35	< 0.001	7.98	2.19	< 0.001
		β			β		
Chair Sit and Reach (cm)	Balanced movers - Intermediate movers	0.88	1.18	0.454	3.03	1.85	0.102
	Balanced movers - Highly sedentary	7.56	1.54	< 0.001	10.76	2.36	< 0.001
	Intermediate movers - Highly sedentary	6.68	1.42	< 0.001	7.73	2.22	< 0.001
		TR			TR		
8-Foot Up and Go (s)	Balanced movers - Intermediate movers	0.96	0.04	0.255	1.03	0.045	0.546
	Balanced movers - Highly sedentary	0.57	0.04	< 0.001	0.62	0.043	< 0.001
	Intermediate movers - Highly sedentary	0.59	0.04	< 0.001	0.61	0.040	< 0.001
		β			β		
6-Minute Walk (m/6 min)	Balanced movers - Intermediate movers	57.07	10.15	< 0.001	−15.20	16.90	0.369
	Balanced movers - Highly sedentary	179.52	13.24	< 0.001	166.48	21.87	< 0.001
	Intermediate movers - Highly sedentary	122.45	12.20	< 0.001	181.67	20.51	< 0.001
		β			β		
CPF -Score (score)	Balanced movers - Intermediate movers	1.83	0.47	< 0.001	0.75	0.64	0.243
	Balanced movers - Highly sedentary	5.72	0.59	< 0.001	6.56	0.79	< 0.001
	Intermediate movers - Highly sedentary	3.89	0.54	< 0.001	5.81	0.74	< 0.001

*Abbreviations*: *SE* Standard error, *RR* Rate Ratio, *TR* Time Ratio, *CPF* Composite Physical Function

All models were adjusted for age

β coefficients are reported for continuous outcomes (linear models), Rate Ratios (RR) for count outcomes (Poisson models), and Time Ratios (TR) for time-to-complete outcomes (Inverse Gaussian models with log link)

*P*-values were corrected for multiple post-hoc-comparisons using the Holm–Bonferroni method

Statistical significance was set at *p* < 0.05

**Table 3:** Estimated marginal means of physical fitness and physical function outcomes by sex-specific physical behaviour profiles

	“Balanced Movers”			“Intermediate Movers”			“Highly Sedentary”		
*Females*	**EMM**	**SE**	**95% CI**	**EMM**	**SE**	**95% CI**	**EMM**	**SE**	**95% CI**
Handgrip (kg/m^2^)	8.85	0.18	8.50; 9.20	8.79	0.17	8.46; 9.12	8.55	0.24	8.07; 9.03
Chair Stand (rep/30 s)	14.18^**c**^	0.24	13.71; 14.66	13.94^**c**^	0.24	13.48; 14.41	10.28^**a, b**^	0.29	9.73; 10.86
Arm Curl (rep/30 s)	17.07^**c**^	0.26	16.56; 17.60	16.55^**c**^	0.26	16.06; 17.06	12.92^**a, b**^	0.32	12.31; 13.57
Back Scratch (cm)	−12.47^**c**^	0.79	−14.01; −10.92	−13.10^**c**^	0.75	−14.58; −11.61	−22.58^**a, b**^	1.12	−24.78; −20.38
Chair Sit and Reach (cm)	−2.21^**c**^	0.85	−3.88; −0.55	−3.10^**c**^	0.82	−4.71; −1.48	−9.78^**a, b**^	1.14	−12.02; −7.53
8 Foot Up-and -go (s)	7.14^**c**^	0.20	6.76; 7.55	7.46^**c**^	0.21	7.06; 7.89	12.55^**ab**^	0.65	11.35; 13.89
6 min Walk (m/6 min)	450.11^**b, c**^	7.31	435.75; 464.46	393.03^**a, c**^	7.09	379.11; 406.96	270.59^**a, b**^	9.86	251.22; 289.95
CPF – score (score)	19.38^**b, c**^	0.35	18.70; 20.06	17.55^a,**c**^	0.32	16.92; 18.18	13.66^**a, b**^	0.43	12.82; 14.49

*Males*	**EMM**	**SE**	**95% CI**	**EMM**	**SE**	**95% CI**	**EMM**	**SE**	**95% CI**
Handgrip (kg/m^2^)	11.81	0.35	11.12; 12.50	12.10^c^	0.30	11.51; 12.68	10.66^b^	0.45	9.79; 11.54
Chair Stand (rep/30 s)	15.11^**c**^	0.39	14.36; 15.89	15.06^**c**^	0.35	14.39; 15.75	10.61^**a, b**^	0.44	9.79; 11.51
Arm Curl (rep/30 s)	18.91^**c**^	0.43	18.08; 19.77	17.99^**c**^	0.37	17.27; 18.74	13.75^**a, b**^	0.49	12.81; 14.75
Back Scratch (cm)	−20.73	1.37	−23.43; −18.04	−18.12^**c**^	1.18	−20.44; −15.80	−26.10^b^	1.82	−29.68; −22.52
Chair Sit and Reach (cm)	−5.41^**c**^	1.39	−8.15; −2.66	−8.44^**c**^	1.24	−10.87; −6.00	−16.16^**a, b**^	1.81	−19.72; −12.61
8 Foot Up-and -go (s)	6.44^**c**^	0.22	6.03; 6.88	6.28^**c**^	0.19	5.92; 6.65	10.33^**ab**^	0.59	9.24; 11.56
6 min Walk (m/6 min)	478.82^**c**^	12.79	453.64; 503.99	494.01^**c**^	11.27	471.84; 516.19	312.34^**a, b**^	16.81	279.25; 345.42
CPF - score	21.60^c^	0.49	20.63; 22.56	20.85^c^	0.42	20.02; 21.68	15.04^a, b^	0.59	13.88; 16.20

*Abbreviations*: *EMM* Estimated marginal mean, *SE* Standard error, *CI* Confidence intervals

All models were adjusted to age

ᵃ difference from “Balanced Movers” profile

^b^ difference from “Intermediate Movers” profile

^c^ difference from “Highly Sedentary” profile

The significance level was set at *p* < 0.05

#### Males

A similar pattern of findings was observed for males, where physical behaviour profiles were significantly associated with all assessed physical fitness and physical function measures. As with females, male “Balanced Movers” and “Intermediate Movers” consistently outperformed the “Highly Sedentary” group. For lower body strength, both “Balanced Movers” and “Intermediate Movers” performed 1.42 times, or 42% more chair stand repetitions (RR = 1.42, *p* < 0.001) than their sedentary peers (Table 2), yielding EMMs of 15.11 and 15.06 repetitions, respectively, versus 10.61 (Table 3). For upper body strength, “Balanced Movers” performed 1.37 times more (RR = 1.37, *p* < 0.001) and “Intermediate Movers” 1.31 times more (RR = 1.31, *p* < 0.001) arm curl repetitions (Table 2), corresponding to means of 18.91 and 17.99 repetitions, compared to 13.75 for their sedentary peers (Table 3). Unlike in females, handgrip strength in males was associated with behaviour profile, with “Intermediate Movers” showing significantly greater strength (β = 1.43, *p* = 0.026) than the “Highly Sedentary” group (Table 2). The “Balanced Movers” and “Intermediate Movers” profiles also exhibited superior flexibility, with advantages of up to 7.98 cm in upper body (β = 7.98, *p* < 0.001) and 10.76 cm in lower body (β = 10.76, *p* < 0.001) flexibility compared to the “Highly Sedentary” group (Table 2). Agility was similarly better, with “Balanced Movers” showing 38% (TR = 0.62, *p* < 0.001) and “Intermediate Movers” 39% (TR = 0.61, *p* < 0.001) faster completion times in the 8-Foot Up and Go test (Table 2). Their mean times were 6.44 s and 6.28 s, respectively, far quicker than the 10.33 s recorded for the “Highly Sedentary” group (Table 3). In the 6-Minute Walk Test, “Balanced Movers” covered 166.48 m more distance (β = 166.48, *p* < 0.001) and “Intermediate Movers” 181.68 m more (β = 181.68, *p* < 0.001) than “Highly Sedentary” males (Table 2). Lastly, their CPF scores were 6.56 points (β = 6.56, *p* < 0.001) and 5.81 points (β = 5.81, *p* < 0.001) higher, respectively, resulting in mean scores of 21.60 and 20.85 compared to 15.04 for the sedentary group (Table 3). However, in contrast to the findings for females, no significant differences were identified between the “Balanced Movers” and “Intermediate Movers” groups across any measure in males.

The associations between physical behaviour profiles and each measure of physical fitness and function are also illustrated in supplementary Figures S3 (females) and S4 (males).

## Discussion

This cross-sectional study applied latent profile analysis —a person-centred approach—to identify distinct profiles of accelerometer-measured physical behaviour (SB, LPA, MVPA) among Portuguese older adults and examined their associations with physical fitness and physical function. Three distinct profiles were identified: “balanced movers”, “intermediate movers”, and “highly sedentary” – highlighting significant heterogeneity in how older adults accumulate physical activity and SB throughout the day. Our findings demonstrate that these profiles are significantly associated with physical fitness and physical function, reinforcing the importance of considering the interplay between different intensities of physical activity and SB.

A key finding in our study was that both the “balanced movers” and “intermediate movers” profiles were associated with substantially better physical fitness and physical function compared to the “highly sedentary” profile, even adjusting for age. This aligns with the broad literature emphasizing the benefits of an active lifestyle [3–9]. Interestingly, the “intermediate movers”, despite engaging in less MVPA and more SB than the “balanced movers”, exhibited comparable levels of performance on most physical fitness tests. Although predominantly sedentary, this group accumulated more LPA and spent less time in SB than the “highly sedentary” group. This finding suggests that even modest increases in daily activity – including LPA - may help mitigate some negative health effects associated with prolonged SB. This aligns with evidence from isotemporal substitution studies, which indicate health benefits when SB is replaced with LPA or MVPA [11, 27, 28]. Although our study used behavioural profiles and cannot be directly compared with studies employing different methodologies, our results align with consistent trends observed in other research that support the potential attenuating role of physical activity in reducing sedentary-related health risks [29].

Our use of latent profile analysis provides insights potentially obscured by variable-centred analyses. While traditional studies might focus solely on whether MVPA guidelines are met, latent profile analysis reveals that the distribution of time across physical behaviour spectrum matters. Other studies using latent profile analysis on physical behaviours or broader lifestyle factors in adults also revel heterogeneous profiles. Although direct comparison is limited by different variables, age, and population, the association of more active profiles with better health outcomes (physical fitness/function in our study; cognitive function, metabolic risk, quality of life and intrinsic capacity in others) is also a recurring theme, strengthening the validity of those behaviour patterns as health determinants [17–20]. Future research could benefit from harmonizing key behaviour indicators to improve comparability across studies.

In our sample, participants with “highly sedentary” profile were, on average, older than those with “balanced movers” or “intermediate movers” profiles, and none met the recommended levels of MVPA (≥ 21.4 min/day). This observation aligns with previous studies showing that older adults are more prone to adopting a sedentary lifestyle and are less inclined to adhere to physical activity recommendations, with this trend becoming more pronounced as age increases [30]. The poorer physical fitness and function in this group, independent of age, underscores the potent negative impact of prolonged inactivity.

Interestingly, while most outcomes differed significantly between “balanced/intermediate movers” and “highly sedentary” profiles, handgrip strength did not differ among female profiles. This observed sex difference in the associations between physical behaviour profiles and handgrip strength, warrants further discussion since it might suggest that physical behaviour patterns, as captured here, have less influence on handgrip strength in older women, or that handgrip strength is influenced by other factors not captured in the profiles. Previous research has demonstrated an indirect effect of MVPA, mediated through muscle power, on lower body strength, agility/dynamic balance, and aerobic endurance in older women [31]; however, this mediating effect has not been explored for upper limb parameters. Moreover, studies in younger populations have identified sex-specific differences in how physical activity — as well as biological factors independent of physical activity — relate to musculoskeletal parameters [32, 33]. The lack of significant difference in handgrip strength could indeed reflect a floor effect. While handgrip strength is a useful proxy for overall muscle strength, its sensitivity may vary by sex and population characteristics, particularly in older women who generally exhibit lower and less variable handgrip strength values. This is supported by findings that handgrip strength has limited contribution as a marker for sarcopenia risk compared to other functional tests such as lower limb muscle power, especially among older females [34]. Therefore, further investigation is warranted, particularly given the importance of handgrip strength in physical fitness assessment and its widespread use to screen for sarcopenia risk [35].

Although our analysis revealed that individuals with a profile classified as “balanced movers” and “intermediate movers” demonstrated higher levels of physical fitness and physical function when compared to those categorized as “highly sedentary”, it’s crucial to note that a substantial proportion of participants in these more active profiles did not meet the recommended fitness standards deemed necessary for maintaining long-term functional independence as established by Rikli and Jones [23]. These standards were established to determine the fitness level necessary for each 5-year age interval to maintain physical independence until age 90 and beyond, despite the expected age-related declines. For example, among individuals with a profile characterised as “balanced movers”, 77.9% of females and 67.6% of males achieved healthy upper body strength cut points, and 70.7% and 64.1% met the health values for lower body strength, respectively. This percentage was slightly lower for “intermediate movers”, with 68.1% of females and 66.7% of males meeting the recommended values for upper body strength, and 64.4 and 66.7 meeting the standards for lower body strength, respectively. When considering other measures of physical fitness, the percentage of individuals with a "balanced movers" or "intermediate movers" profiles meeting the expected health benchmarks dropped to as low as 50%. These findings highlight that even older adults with relatively more active profiles require targeted interventions to improve specific fitness domains. Regarding the participants with a "highly sedentary" profile, more than 90% of both, females and males showed risk values for aerobic endurance assessed by 6 min of walk or values for agility, assessed by 8-ft up-and-go. According to physical function scores assessed using the CPF scale, around 74% of “highly sedentary” females and 61% of males may be at risk of losing physical independence.

Strengths of this study include the measurement of physical behaviours using accelerometry, the comprehensive assessment of physical fitness and function, and the application of a person-centred analytical approach using latent profile analysis to identify physical behaviour patterns which allows for nuanced insights that traditional variable-centred approaches may overlook. This type of analysis can consider the combined effect of the range of physical activity intensities and SB, offering a more integrated and comprehensive understanding of physical behaviours. However, several limitations must be acknowledged. First, the cross-sectional design precludes establishing causality. While we observed associations, longitudinal studies are needed to confirm if behaviour profiles predict future declines in physical fitness and physical function. Second, accelerometry has limitations, it may not accurately capture certain activities (e.g., cycling, resistance training and water-based exercises), which could potentially underestimate physical activity levels. Third, our analysis adjusted only for age. Other potential confounders, such as socio-economic status, comorbidities, and dietary habits were not included in the models and could influence the observed associations. Future studies should aim to incorporate a wider range of covariates. Fourth, the findings are specific to Portuguese community-dwelling older adults, which may limit generalizability to other populations.

Despite limitations, our findings have practical implications. They highlight the potential benefits of interventions targeting reductions in SB and increases in LPA, particularly for the segment of older adults who are “highly sedentary”. Latent profile analyses could potentially be used to tailor interventions; for example, “highly sedentary” individuals might initially benefit most from strategies focused solely on reducing SB, while “intermediate movers” might be encouraged to gradually incorporate more MVPA. Future research should employ longitudinal designs to better understand how behavioural profiles evolve over time and how these changes influence physical fitness and function. Investigating the determinants of profile membership (e.g., psychosocial factors, environmental influences) is also crucial. Incorporating other behaviours, such as sleep time and domain-specific SB, into latent profile analysis models could provide a more comprehensive understanding of 24-hour movement patterns. Finally, intervention studies designed based on latent profile could test the effectiveness of tailored approaches to promote healthier behavioural patterns in older adults.

## Conclusion

Using a latent profile analysis (a person-centred approach), which account for the interplay of SB and physical activity intensities, this study identified three distinct physical behaviour profiles among Portuguese older adults. Profiles characterized by lower SB and higher engagement in physical activity, were associated with better physical fitness and function, even when MVPA recommendations were not fully met. While achieving recommended levels of MVPA is ideal, these findings underscore the detrimental impact of high SB and highlight the importance of reducing sedentary behaviour and promoting LPA along with MVPA for preserving functional health in ageing. By uncovering these behavioural profiles among older adults, latent profile analysis provides valuable insights to guide the development of more personalized interventions for healthy ageing.

## Supplementary Information

Supplementary Material 1.

## Data Availability

The data that support the findings of this study are available from the corresponding author upon reasonable request.

## Abbreviations

ADLs: Activities of daily living
SB: Sedentary behaviour
LPA: Light physical activity
MVPA: Moderate-to-vigorous physical activity
CPF: Composite Physical Function
BMI: Body Mass Index
AIC: Akaike Information Criterion
BIC: Bayesian Information Criterion
