# Supplementary material for: Physical behaviour profiles and their associations with fitness and function in older adults: a cross-sectional latent profile analysis
Source: Eur Rev Aging Phys Act. 2026 Jan 12;23:7. doi: 10.1186/s11556-025-00397-4 (PMC12888268; doi:10.1186/s11556-025-00397-4)
Supplement: Supplementary file 1 — Supplementary Material 1. [file 11556_2025_397_MOESM1_ESM.docx]

**Physical Behaviour Profiles and Their Associations with Fitness and Function in Older Adults: A Cross-Sectional Latent Profile Analysis**

Table S1 - Females Physical behaviour class comparisons

| **Class** | **LogLik** | **AIC** | **AWE** | **BIC** | **CAIC** | **CLC** | **KIC** | **SABIC** | **ICL** | **Entropy** |
| --- | --- | --- | --- | --- | --- | --- | --- | --- | --- | --- |
| 1 | -7814.46 | 15640.91 | 15724.59 | 15668.75 | 15674.75 | 15630.91 | 15649.91 | 15649.70 | -15668.75 | 1.00 |
| 2 | -7108.81 | 14243.62 | 14427.43 | 14303.94 | 14316.94 | 14219.45 | 14259.62 | 14262.66 | -14351.02 | 0.91 |
| 3 | -6837.67 | 13715.33 | 13999.21 | 13808.13 | 13828.13 | 13677.05 | 13738.33 | 13744.62 | -13923.25 | 0.86 |
| 4 | -6671.49 | 13396.98 | 13780.77 | 13522.25 | 13549.25 | 13344.74 | 13426.98 | 13436.52 | -13641.67 | 0.88 |
| 5 | -6576.47 | 13220.95 | 13704.72 | 13378.70 | 13412.70 | 13154.69 | 13257.95 | 13270.74 | -13527.48 | 0.87 |
| 6 | -6449.38 | 12980.75 | 13564.46 | 13170.99 | 13211.99 | 12900.51 | 13024.75 | 13040.79 | -13320.47 | 0.88 |

Figure S1 – Female - Elbow Plot Showing Physical Behaviour Class Comparisons


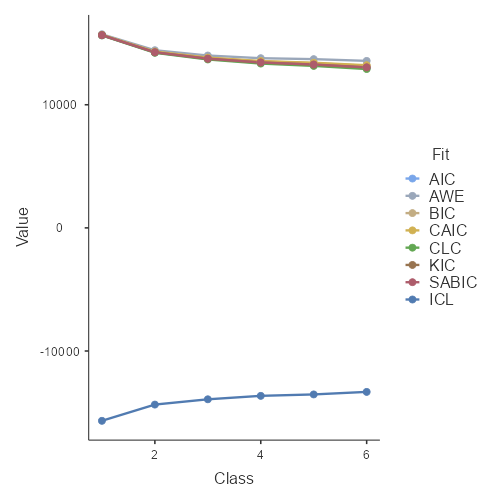


Abbreviations: LogLik (Log-likelihood); AIC (Akaike Information Criterion); AWE (Approximate Weight of Evidence); BIC (Bayesian Information Criterion); CAIC (Consistent AIC); CLC (Classification Likelihood Criterion); KIC (Kullback Information Criterion); SABIC (Sample Size Adjusted BIC); ICL (Integrated Completed Likelihood)

Table S2 – Males Physical behaviour class comparisons

| Class | LogLik | AIC | AWE | BIC | CAIC | CLC | KIC | SABIC | ICL | Entropy |
| --- | --- | --- | --- | --- | --- | --- | --- | --- | --- | --- |
| 1 | -3458.97 | 6929.94 | 7003.53 | 6952.74 | 6958.74 | 6919.94 | 6938.94 | 6933.70 | -6952.74 | 1.00 |
| 2 | -3230.82 | 6487.65 | 6649.75 | 6537.03 | 6550.03 | 6463.32 | 6503.65 | 6495.80 | -6573.27 | 0.84 |
| 3 | -3078.92 | 6197.84 | 6448.09 | 6273.82 | 6293.82 | 6159.55 | 6220.84 | 6210.38 | -6322.54 | 0.86 |
| 4 | -3052.78 | 6159.57 | 6498.07 | 6262.14 | 6289.14 | 6107.22 | 6189.57 | 6176.50 | -6338.10 | 0.82 |
| 5 | -2969.39 | 6006.78 | 6433.40 | 6135.95 | 6169.95 | 5940.49 | 6043.78 | 6028.10 | -6203.47 | 0.86 |
| 6 | -2928.55 | 5939.10 | 6453.89 | 6094.87 | 6135.87 | 5858.84 | 5983.10 | 5964.81 | -6163.80 | 0.87 |

Figure S2 – Males - Elbow Plot Showing Physical Behaviour Class Comparisons


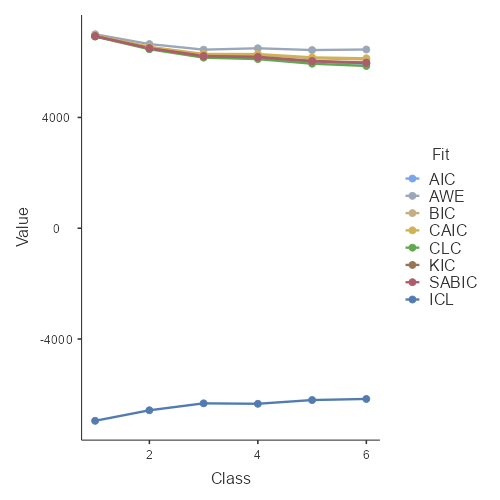


Abbreviations: LogLik (Log-likelihood); AIC (Akaike Information Criterion); AWE (Approximate Weight of Evidence); BIC (Bayesian Information Criterion); CAIC (Consistent AIC); CLC (Classification Likelihood Criterion); KIC (Kullback Information Criterion); SABIC (Sample Size Adjusted BIC); ICL (Integrated Completed Likelihood)

Figure S3 - Females -Association of physical behaviour profiles on each measure of physical fitness and physical function controled to age.


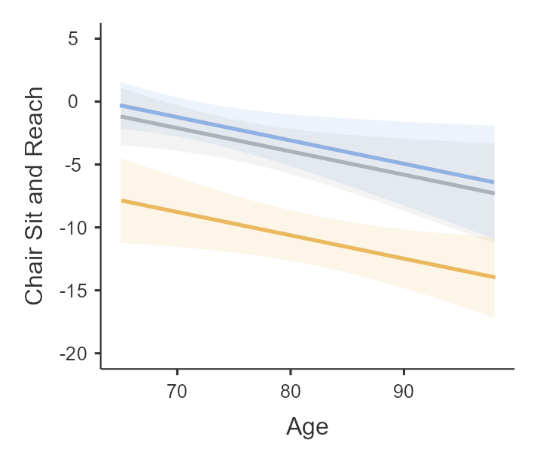

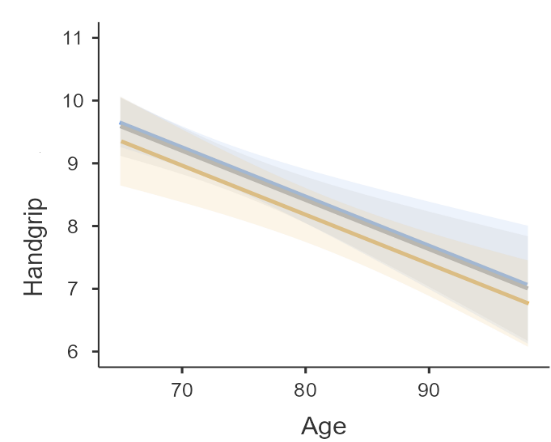


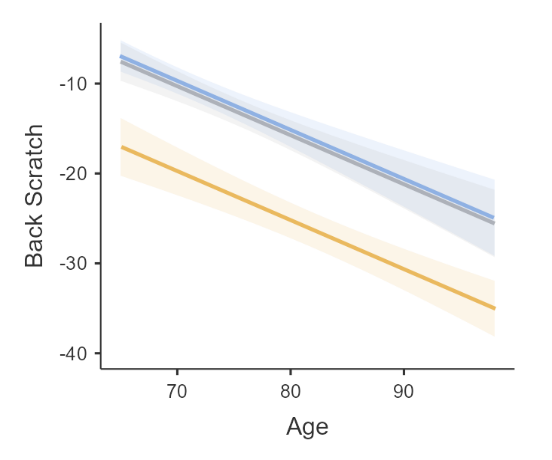

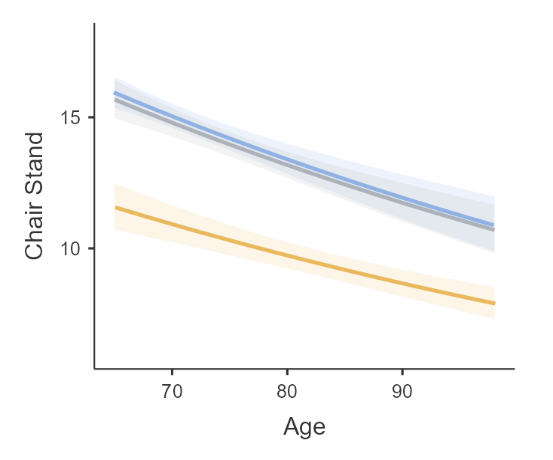


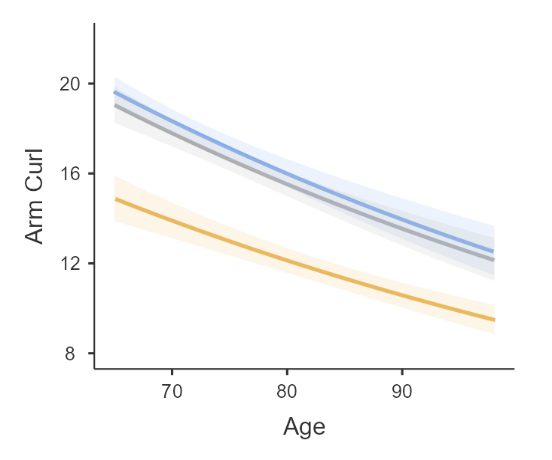


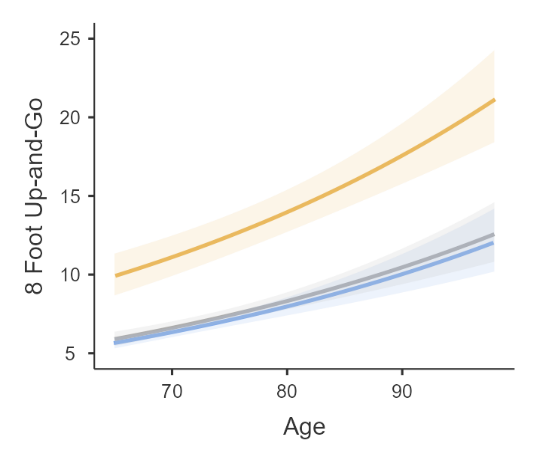


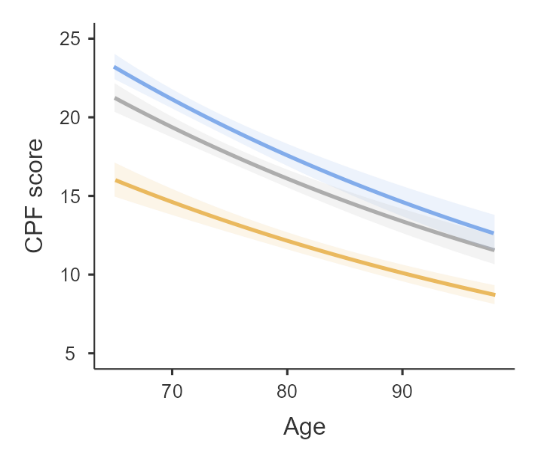

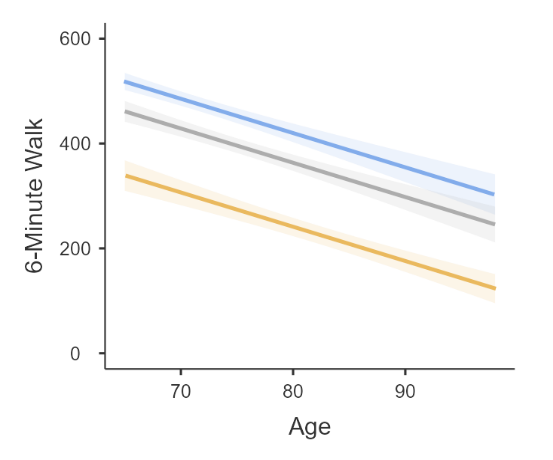


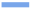
Balanced movers
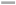
 Intermediate movers
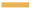
 Highly sedentary


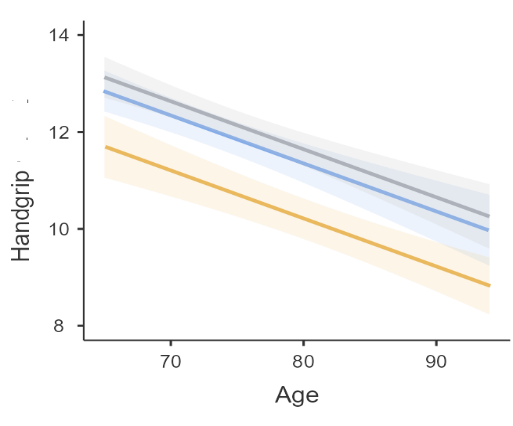
Figure S4 - Males - Association of physical behaviour profiles on each measure of physical fitness and physical function controled to age


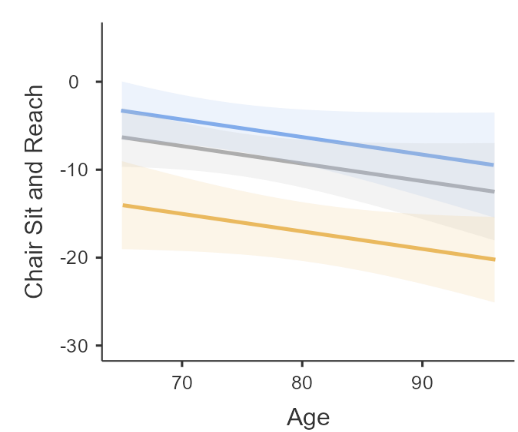


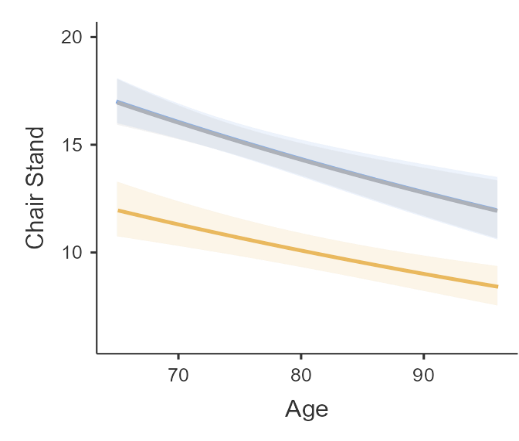


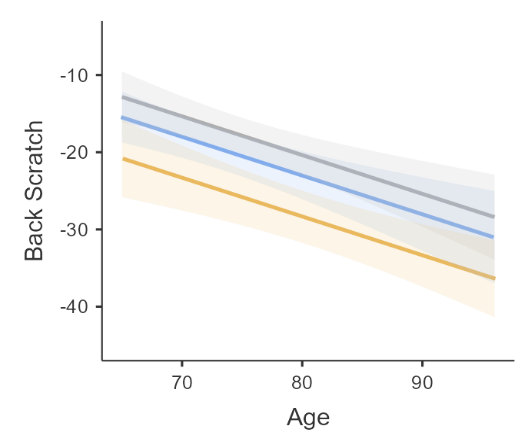


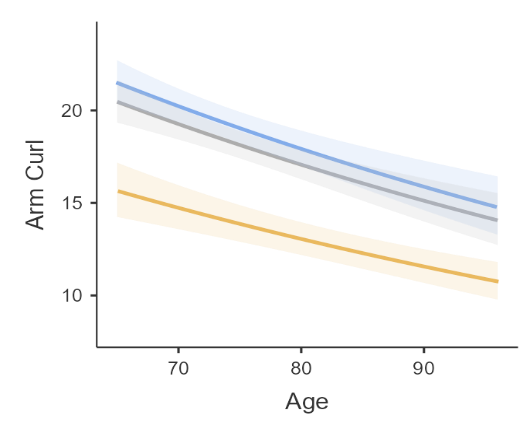


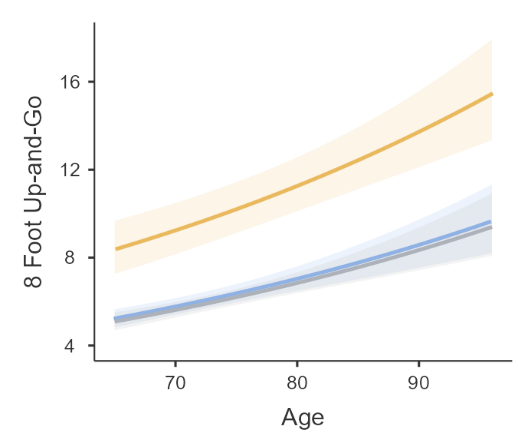


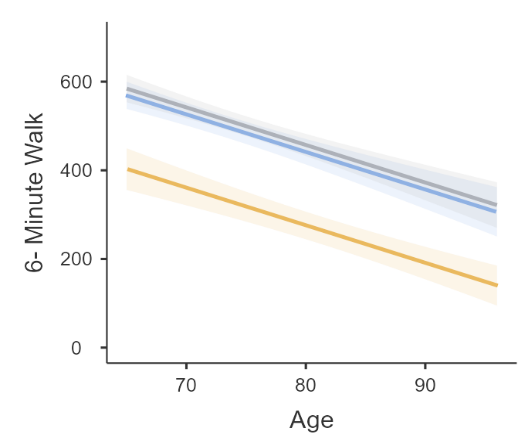


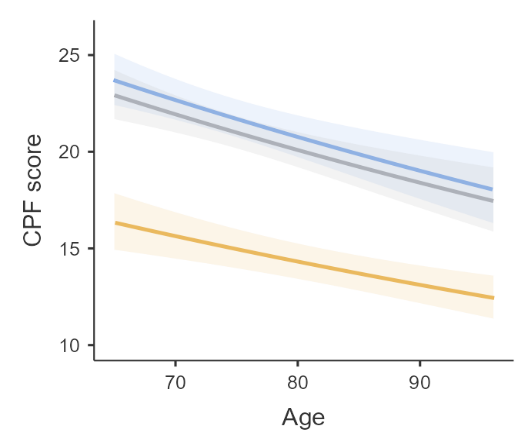


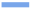
Balanced movers
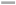
 Intermediate movers
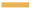
 Highly sedentary
